# No staghorn calculi and none/mild hydronephrosis may be risk factors for severe bleeding complications after percutaneous nephrolithotomy

**DOI:** 10.1186/s12894-021-00866-9

**Published:** 2021-08-13

**Authors:** Xue Dong, Dongnv Wang, Huangqi Zhang, Shuzong You, Wenting Pan, Peipei Pang, Chaoqian Chen, Hongjie Hu, Wenbin Ji

**Affiliations:** 1 Department of Radiology, Taizhou Hospital, Zhejiang University, Taizhou, 318000 Zhejiang China; 2 Department of Urology, Taizhou Hospital of Zhejiang Province, Taizhou, 318000 Zhejiang China; 3 Department of Radiology, School of Medicine, Sir Run Run Shaw Hospital, Zhejiang University, Hangzhou, 310023 Zhejiang China; 4 Department of Pharmaceuticals Diagnosis, GE Healthcare, Hangzhou, China

**Keywords:** Percutaneous nephrolithotomy, Postoperative complications, Staghorn calculi, Hydronephrosis, The modified Clavien scoring, Computed tomography

## Abstract

**Background:**

To explore the risk factors for severe bleeding complications after percutaneous nephrolithotomy (PCNL) according to the modified Clavien scoring system.

**Methods:**

We retrospectively analysed 2981 patients who received percutaneous nephrolithotomies from January 2014 to December 2020. Study inclusion criteria were PCNL and postoperative mild or severe renal haemorrhage in accordance with the modified Clavien scoring system. Mild bleeding complications included Clavien 2, while severe bleeding complications were greater than Clavien 3a. It has a good prognosis and is more likely to be underestimated and ignored in retrospective studies in bleeding complications classified by Clavien 1, so no analysis about these was conducted in this study. Clinical features, medical comorbidities and perioperative characteristics were analysed. Chi-square, independent *t* tests, Pearson’s correlation, Fisher exact tests, Mann–Whitney and multivariate logistic regression were used as appropriate.

**Results:**

Of the 2981 patients 70 (2.3%), met study inclusion criteria, consisting of 51 men and 19 women, 48 patients had severe bleeding complications. The remaining 22 patients had mild bleeding. Patients with postoperative severe bleeding complications were more likely to have no or slight degree of hydronephrosis and have no staghorn calculi on univariate analysis (*p* < 0.05). Staghorn calculi (OR, 95% CI, *p* value 0.218, 0.068–0.700, 0.010) and hydronephrosis (OR, 95% CI, *p* value 0.271, 0.083–0.887, 0.031) were independent predictors for severe bleeding via multivariate logistic regression analysis. Other factors, such as history of PCNL, multiple kidney stones, site of puncture calyx and mean corrected intraoperative haemoglobin drop were not related to postoperative severe bleedings.

**Conclusions:**

The absence of staghorn calculi and a no or mild hydronephrosis were related to an increased risk of post-percutaneous nephrolithotomy severe bleeding complications.

**Supplementary Information:**

The online version contains supplementary material available at 10.1186/s12894-021-00866-9.

## Background

Percutaneous nephrolithotomy (PCNL) is the primary choice for the treatment of larger than 2 cm renal stones [1]. PCNL, which is a commonly applied operation, has a lower complication rate than open surgery. However, severe bleeding after percutaneous nephrolithotomy is still a challenge for urologists. Different research groups have evaluated the potential impact of several clinical, radiological, and perioperative variables on postpercutaneous nephrolithotomy bleeding [2, 3].

Unfortunately, because there is no general consensus on the definition of bleeding complications [4], few published reports have applied specific bleeding complication scores to urological surgery. Surgery, including urology, has used the Clavien-Dindo classification system extensively [5, 6] due to its simplicity and usability in classifying postoperative complications [7]. Postoperative complications after urological surgery were recommended to be reported by the European Association of Urology (EAU) guidelines panel, and the Clavien-Dindo classification system was the primary choice [8]. In 2012 de la Rosette et al. found that urologists tended to have a lower rate of agreement for grading minor complications (Clavien score 1) and best agreement was identified to define severe complications (Clavien greater than 3a) [9].

Past analysis of prognostic factors showed that puncture correctness [2], blood transfusion [3], puncture via the inferior calyx, and multiple or isolated kidney stones [10] were previously reported to be independently associated with severe bleeding after PCNL. Severe postoperative bleeding complications are a challenge to urologists and patients. To the best of our knowledge, no studies have investigated the factors associated with severe bleeding complications based on the modified Clavien score. The goal of our work was to identify factors associated with severe bleeding complications after PCNL according to the modified Clavien score [9]. We determined the clinical, radiological, and perioperative features that were related to severe bleeding complications after PCNL.

## Methods

### Study design and patients

This retrospective research was approved by the institutional review board. The requirement to obtain patient consent was waived. The study analysed the data of 2,981 patients who received PCNL at Taizhou Enze Medical Center (Group) Taizhou Hospital from January 2014 to December 2020. Cases with missing data and no significant complications related to bleeding were excluded. Only patients with complete clinical data who underwent unenhanced abdominal computed tomography (CT) (slice thickness 5 mm) to assess renal stone information and nephrosonography to evaluate hydronephrosis preoperatively were included.

### Clinical data

The information collected included demographics, clinical and perioperative characteristics and renal stone characteristics. Demographic features included age, sex, and BMI. The clinical characteristics assessed were clinical characteristics (urinary tract infections, haematuria and pain), preoperative creatinine, history of anticoagulation, medical comorbidities (hypertension, diabetes mellitus, ischaemic heart disease, chronic lung disease, cardiorespiratory diseases, and chronic kidney disease), open surgery history and a previous history of PCNL. Renal stone features included laterality, stone location, stone count, peak Hounsfield unit, and the presence of staghorn calculi. Staghorn calculi is defined any branched stone occupying more than one portion of the collecting system [11]. Hydronephrosis was graded as either none/mild or moderate/severe using the Society of Fetal Urology grading system [12, 13]. Perioperative characteristics were the calyx of access, dilatation type, operative time (OT) and corrected intraoperative haemoglobin drop. Haemoglobin levels were corrected (corrected haemoglobin drop) for any prior transfusion. One unit of packed red blood cells or whole blood transfused was defined as the equivalent of 10 g/L haemoglobin drop [14].

**Fig. 1:**
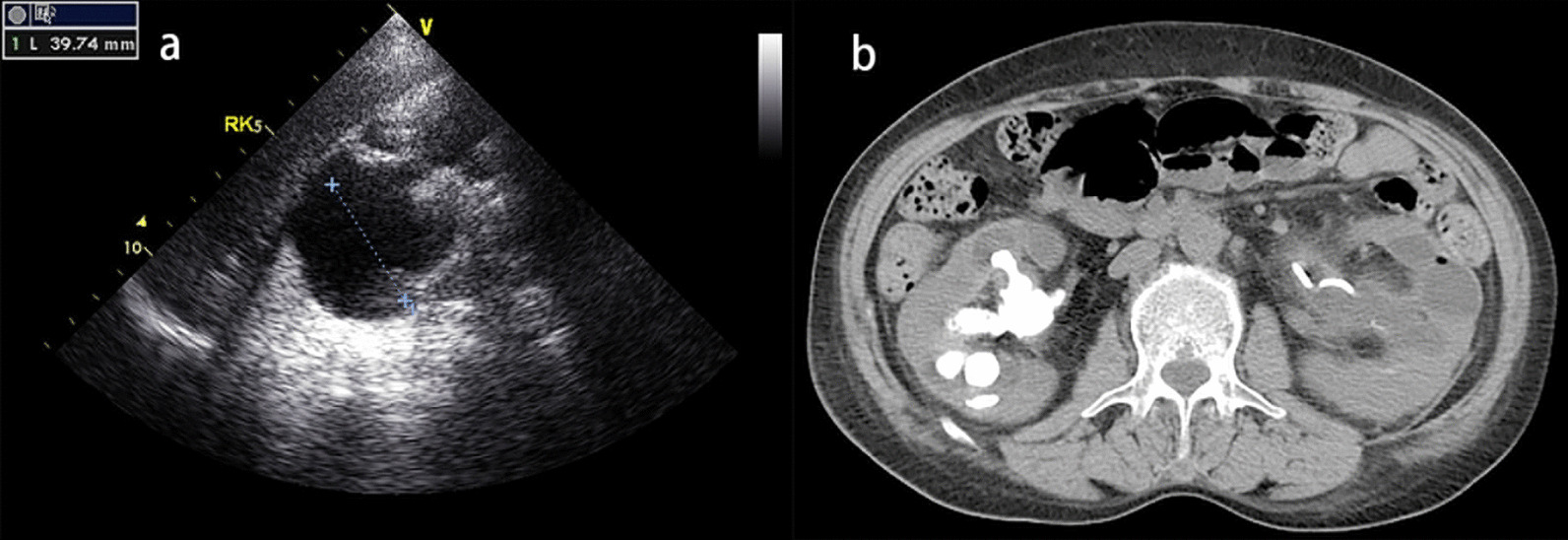
Mild complication after PCNL in 57-year-old woman who underwent CT scan and nephrosonography preoperatively. **a** Sonagraphic view demonstrated anteroposterior diameter (APD) of right kidney was 39.74 mm, severe hydronephrosis. **b** CT view of staghorn calculi

**Fig. 2:**
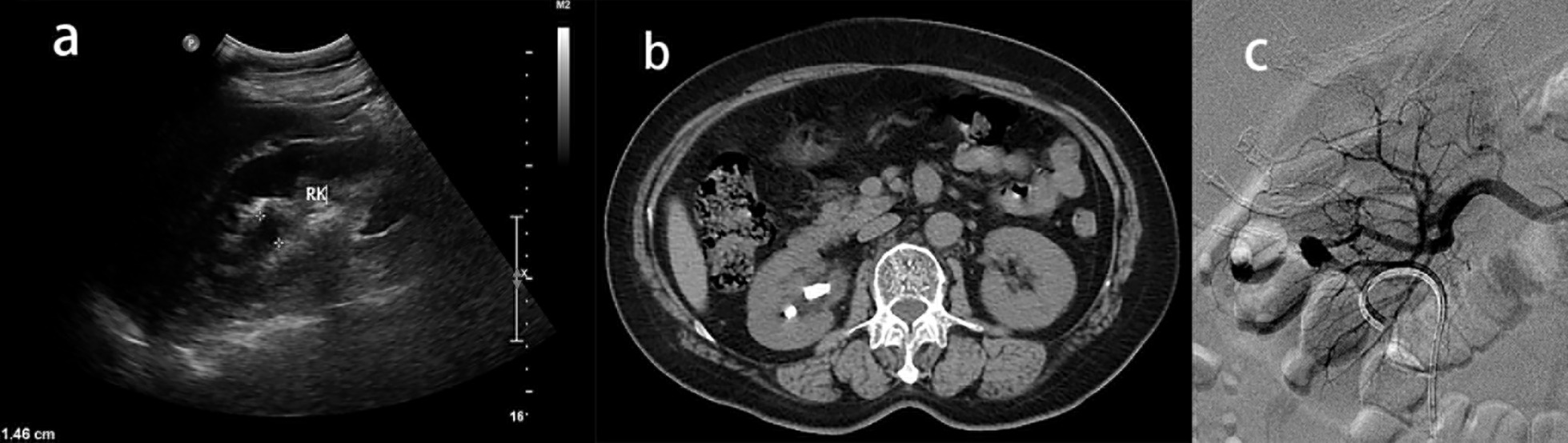
Severe complication after PCNL in 56-year-old woman who underwent CT scan and nephrosonography preoperatively. **a** Sonagraphic view demonstrated anteroposterior diameter (APD) of right kidney was 14.6 mm, mild hydronephrosis. **b** CT view of no staghorn calculi. **c** Superselective renal arteriogram showed extravasation in right kidney mid segmental branches

Bleeding complications were collected during postoperative follow-up and categorized according to the modified Clavien score. It has a lower rate of agreement for grading minor complications (Clavien score 1) [9] and a good prognosis, so no analysis about these was conducted in this study. Major bleeding complications were defined as greater than Clavien 2. Mild bleeding complications in this study were defined as Clavien 2, and severe bleeding were classified as Clavien 3a, 3b, 4a, 4b and 5. When patients had more than 1 complication, the final analysis included only the highest Clavien score (Additional file 1: Table S1). Examples of views are shown for a mild bleeding complication undergoing blood transfusion in Fig. 1 and a severe bleeding complication undergoing a superselective renal arteriogram in (Fig. 2).

### Surgical technique

PCNL procedures were performed according to the local clinical guidelines and practices. After placing the patient in the lithotomy position, retrograde ureter catheterization was performed. All other procedures were completed in the prone position. The standard technique involved using ultrasound and/or fluoroscopy in obtaining percutaneous renal access. After obtaining renal access, the tract was dilated using either balloon dilation or Amplatz serial dilation before placement of an access sheath and nephroscope. Amplatz dilatation or balloon dilatation were used according to indications on the patients. An intracorporeal lithotripsy device was then introduced through the working channel of the nephroscope and stones were broken down using a pneumatic lithotripter and/or holmium laser and then stone fragments were removed. At the end of the procedure, the system may be examined with a flexible endoscope. The procedure was considered to have been completed when all removable stones have been taken out. Internal and/or and external drain(s) were positioned according to the judgment of the surgeon.

### Statistical analysis

Statistical analysis was performed with IBM SPSS (version 25; IBM, New York, USA). To evaluate significant factors for severe bleeding after PCNL, chi-square, Fisher’s exact tests were used for the categorical variables. The independent *t*-test, Pearson correlation, and Mann–Whitney *U* test were used for the analysis of quantitative variables. To identify predictive factors associated with severe bleeding, multivariable logistic regression was used, and the corresponding odds ratios (ORs) and 95% confidence intervals (CIs) were determined. All *p* values were two-tailed, and a *p* value of < 0.05 was considered statistically significant.

## Results

Of the 2981 patients who received PCNL, In accordance with the modified Clavien score [15], the mild bleeding group included 22 patients (22 blood transfusions), and the severe bleeding group included 48 patients (45 angioembolization, 2 multiple bladder washouts, 1 nephrectomy and ICU management). The mean ± SD age of 70 patients was 57.5 ± 13.7, and men accounted for 72.9%. The mean ± SD BMI was 22.6 ± 3.1 kg/m^2^. According to our preoperative assessment, 62.9% of the cases suffered from flank pain, 7.1% of patients presented with a history of haematuria, and 92.9% had a history of previous urinary tract infections. Hypertension and chronic renal disease, accounting for 42.9% and 32.9% of the cohort, were two of the most common comorbidities. A total of 4.3% of the patients had a history of using anticoagulant medication.

A preoperative test revealed an IQR serum creatinine of 81.5 μmol/L (67.8–127.5). According to the preoperative imaging, 50.0% of patients of major bleeding had staghorn calculi. A total of 57.1% of the patient’s calculi were located in the left kidney, and multiple calculi were present in 71.4% of the cases. A total of 71.4% of the patients had no hydronephrosis or only a mild degree of hydronephrosis. A total of 28.6% of the patients presented with a mid or severe degree of hydronephrosis. The pole access was used the upper calyx in 0 cases, the middle calyx in 63 cases, the lower calyx in 1 cases and multiple calyx the upper in 6 cases. The average corrected intraoperative haemoglobin drop from the pre-PCNL to the first post-PCNL day was 20.6 ± 15.2 g/dL (range 0–68). The mean OT was 108.5 ± 39.9 (range 31–236) minutes. Occurrence times of management strategy of the highest Clavien grade in final analysis were ranged from 1 to 24 days after PCNL.

Table 1 shows a comparison of the patient demographics, preoperative clinical characteristics and renal stone characteristics between mild bleeding complications and severe bleeding complications. Staghorn calculi (*p* = 0.010) and the degree of hydronephrosis (*p* = 0.034) were significantly related to severe bleeding complications in univariate analysis. None of perioperative characteristics were significantly associated with severe bleeding complications in Table 2. Multivariate analysis showed that only the absence of staghorn stones (OR, 95% CI, *p* value 0.218, 0.068–0.700, 0.010) and none or mild degree of hydronephrosis (OR, 95% CI, *p* value 0.271, 0.083–0.887, 0.031) were a higher risk of severe bleeding in Table 3. The mild and severe bleeding complications are shown in Table 4. By the modified Clavien score. 45 cases’ bleeding complications were managed by angioembolization and their angiographic findings are shown in Table 5.

**Table 1:** Patient demographic, preoperative clinical characteristics and renal stone characteristics

No. pts (%)	Major bleeding complication	Mild bleeding complication	Severe bleeding complication	*p*
	70	22	48	
Sex (%)				
Male	51 (72.9)	14 (63.6)	37 (77.1)	0.240
Female	19 (27.1)	8 (36.4)	11 (22.9)	
Age (years, mean ± SD)	57.5 (13.7)	59.3 (13.6)	56.7 (13.8)	0.471
BMI (kg/m^2^, mean ± SD)	22.6 (3.1)	21.8 (3.1)	23.0 (3.0)	0.139
Preop creatinine (μmol/L, IQR)	81.5 (67.8–127.5)	83.0 (70.5–163.8)	80.5 (67.0–124.0)	0.494
History of anticoagulation (%)	3 (4.3)	2 (9.1)	1 (2.1)	0.231
Preop clinical (%)				
Pain	44 (62.9)	12 (54.5)	32 (66.7)	0.330
Hematuria	5 (7.1)	1 (4.5)	4 (8.3)	1.000
UTIs	65 (92.9)	20 (90.9)	45 (93.8)	0.646
Preop. comborbidities (%)				
Hypertension	30 (42.9)	12 (54.5)	18 (37.5)	0.181
Diabetes mellitus	11 (15.7)	4 (19.0)	7 (14.6)	0.725
Ischemic heart disease	14 (20.0)	6 (27.3)	8 (16.7)	0.344
Chronic lung disease	6 (8.6)	2 (9.1)	4 (8.3)	1.000
Chronic kidney disease	23 (32.9)	7 (31.8)	16 (33.3)	0.900
At least 1	48 (68.6)	18 (81.8)	30 (62.5)	0.106
Open surgery history (%)	11 (15.7)	6 (27.3)	5 (10.4)	0.088
History of PCNL (%)	11 (15.7)	5 (22.7)	6 (12.5)	0.303
Calculi side (%)				
Lt	40 (57.1)	11 (50.0)	29 (60.4)	0.414
Rt	30 (42.9)	11 (50.0)	19 (39.6)	
Stone count (%)				
Single	20 (28.6)	7 (31.8)	13 (27.1)	0.684
Multiple	50 (71.4)	15 (68.2)	35 (72.9)	
Stone type (%)				
Staghorn^a^	35 (50.0)	16 (72.7)	19 (39.6)	0.010
Others	35 (50.0)	6 (27.3)	29 (60.4)	
peak Hounsfield unit (Hu, IQR)	1095.0 (735.0–1406.0)	1282.0 (723.5–1506.3)	1086.0 (710.0–1334.0)	0.425
Hydronephrosis (%)				
None or mild	50 (71.4)	12 (54.5)	38 (79.2)	0.034
Moderate or severe	20 (28.6)	10 (45.5)	10 (20.8)	

*UTIs* urinary tract infections, *BMI* body mass index, *IQR* interquartile range

^a^Any branched stone occupying more than one portion of the collecting system

**Table 2:** Perioperative characteristics

No. pts (%)	Major bleeding complication	Mild bleeding complication	Severe bleeding complication	*p*
	70	22	48	
OT (min, mean ± SD)	108.5 (39.9)	119.2 (41.7)	103.4 (38.4)	0.127
Corrected intraoperative hemoglobin drop (g/dL, mean ± SD)	20.6 (15.2)	19.8 (18.0)	20.9 (13.9)	0.785
Dilatation type (%)				
Amplatz	51 (72.9)	16 (72.7)	35 (72.9)	0.987
Balloon	19 (27.1)	6 (27.3)	13 (27.1)	
No. renal accesses (%)				
Single	64 (91.4)	20 (90.9)	44 (91.7)	1.000
Multiple	6 (8.6)	2 (9.1)	4 (8.3)	
Pole access (%)				0.697
Upper calyx	0 (0)	0 (0)	0 (0)	
Mid calyx	63 (90)	19 (86.4)	44 (91.7)	
Lowe calyx	1 (1.4)	1 (4.5)	0 (0)	0.314
Multiple calyx	6 (8.6)	2 (9.1)	4 (8.3)	

*OT* operative time

**Table 3:** Multivariate analysis of severe bleeding complication after percutaneous nephrolithotomy

	OR (95% CI)	*p*
Staghorn	0.218 (0.068–0.700)	0.010
Hydronephrosis	0.271 (0.083–0.887)	0.031

*OR* corresponding odds ratio, *CIs* confidence interval

**Table 4:** Major bleeding complications by modified Clavien score

	Clavien category	No. cases
2	Bleeding requiring blood transfusion	22
3a	Bleeding requiring multiple bladder washouts	2
3b	Bleeding managed by angioembolisation	45
4a	Bleedingrequiring ICU management	1
Total		70

**Table 5:** Angiographic findings in 45 patients

Angiographic findings	No. cases
Contast extravasation PA AVF	26 8 4
PA and AVF	2
PA and Contast extravasation	3
AVF and Contast extravasation	2

*PA* Pseudoaneurysm, *AVF* Arteriovenous fistula

## Discussion

PCNL plays an indispensable role in treating large kidney stones [16]. Since PCNL was popularized in 1976 [15], it has been proven to be a valid procedure for the removal of kidney stones after decades of surgical experience.

Bleeding complications after PCNL are dangerous or even life-threatening. Conservative treatment can be used to control venous haemorrhage, while arterial lesions are rare but require immediate intervention. Several studies have shown that the occurrence of blood transfusions caused by PCNL varies widely, from 1 to 5% [4, 17]. The rate of postoperative angioembolization is not high, ranging from 0 to 1.5% [18]. Due to the simple treatment methods, such as using intravenous fluid without blood transfusion, requiring a single episode of nephrostomy clamping or skin compression/pressure dressing [19], minor bleeding only poses a slight threat to patients and has a lower rate of agreement with urologists [9], and therefore cases of bleeding complications classified as Clavien 1 were not included in this research.

Several studies have identified prognostic factors for severe bleeding complications after PCNL [2, 3, 10, 20]. However, due to the absence of standardized reporting classification systems, their results are difficult to interpret and compare. Sitki et al. [3] showed that the predictors for severe bleeding complications were stone load and renal abnormalities. However, this study evaluated risk factors for post-PCNL bleeding that required embolization from blood transfusion so these cases of direct angioembolization without a blood transfusion have been ignored. Therefore, we evaluated bleeding complications in accordance with the modified Clavien scoring system, which is advised when reporting urological operation outcomes [16, 21].

Severe postoperative bleeding is a challenge to both the patient and the surgeon. Our analysis sought to identify prognostic factors for severe bleeding complications after PCNL in our hospital. In this study, we found that staghorn calculi and the degree of hydronephrosis were related to severe bleeding complications. In multivariate analysis, no staghorn calculi and none or mild hydronephrosis were significant predictors of the severe bleeding.

Staghorn stones represent 10–20% of all nephrolithiasis cases [22]. In our experience, the absence of staghorn calculi is a predictor of severe bleeding post-PCNL. Previous studies have come to inconsistent conclusions. In previous studies, the presence of staghorn stones [23, 24] was a predictor of severe postoperative bleeding. Zehri et al. reported [23] that the occurrence of staghorn stones can significantly predict severe vascular lesions after PCNL. In El-Nahas’s [24] study, vascular embolization was needed in 39 of 3878 patients undergoing PCNL, and multivariate analysis found that staghorn stones were a significant risk factor. One explanation is that staghorn stones may prolong the operative time, leading to an increased chance of bleeding after surgery.

However, the conclusions of these studies were not different from the common bleeding complications reported by other studies after PCNL [17, 25, 26] and were not consistent with the results of our study. One reason for this may be that previous studies have compared outcomes in patients who underwent embolization with those who did not have any bleeding complications, while in our study, risk factors for severe bleeding were only discussed in patients with bleeding complications of greater than Clavien 2 as reported by the modified Clavien scoring system. Another reason for presence of staghorn calculi predicting mild bleeding may be that the patients formating staghorn stones without obvious symptoms leading to medical treatment indicate that the kidneys of them may have a better tolerance than these of no staghorn stones that occur less severe bleeding complication after PCNL.

In this study, mild or absence of hydronephrosis was observed to be associated with an increased risk of severe bleeding complications following PCNL. This result is consistent with the research of Lee et al. [27]: no hydronephrosis was a predictor for severe bleeding after PCNL. Similarly, Hee et al. stated that a lack of hydronephrosis was an important risk factor for transfusion in conventional PCNL [28]. Senocak et al. [13] found that the degree of hydronephrosis was significantly related to the reduction of blood loss during paediatric PCNL. Wang et al. [29] reported that no hydronephrosis occurred in patients with severe renal haemorrhage.

There are several possible explanations for the findings of our study. Patients without moderate or severe hydronephrosis may have a thicker renal parenchyma, which increases the risk of severe bleeding from puncture. Moderate and severe hydronephrosis can lead to thinning of the renal cortex and eventually ischaemia, which may be related to reduced renal blood flow. Another possible explanation is that none or mild hydronephrosis may reduce the availability of calculi and thus requiring more manipulation during the surgery, which may cause larger lesions to renal vessels and parenchyma.

In Sitki’s [3] study, the risk factors were analysed for bleeding complications that required renal arterial embolization and blood transfusion, and the stone load, renal abnormalities, and the mean corrected haemoglobin drop predicted severe bleeding after PCNL. Tan et al. [10] demonstrated that severe haemorrhage after PCNL is related to lower calyx puncture and multiple kidney stones. Diabetes was a predictor of bleeding in both studies [13, 25]. In our study, factors such as BMI, hypertension, diabetes, chronic kidney disease, corrected haemoglobin drop, site of punctured calyx, and multiple stones did not have any significant correlation with severe bleeding. This suggests that these covariates may be predictors for postoperative blood loss in general but not for severe bleeding complications.

The significance of this study is that in patients who have already experienced relatively observable bleeding, those who have no staghorn stones and/or none or mild hydronephrosis preoperatively need to be managed carefully because they are more likely to have severe bleeding complications. PCNL patients with none or mild hydronephrosis may benefit from retrograde intrarenal surgery [30] with a holmium laser and may be ideal candidates for its use. The findings of this study may play a role in the surgical decision-making for patients with renal stones.

Several limitations exist in our study. First, any retrospective study may result in an underestimate of postoperative bleeding complications. However, patients with bleeding complications of greater than Clavien 2 are hardly ignored because further or invasive operations are required, which is why we focused on this group. Second, the sample size of this study was small, which had a certain impact on the results, but samples with severe bleeding are difficult to obtain. Moreover, all PCNLs in our hospital were conducted in the prone position, so these findings may not be applicable to PCNLs performed in the supine position.

## Conclusions

After reviewing our experience with PCNL, we identified predictors of severe bleeding postoperatively. Absence of staghorn stones or no/mild hydronephrosis were independently associated with the high risk of severe bleeding. In patients with obvious bleeding, it is necessary to take extreme precautions for those with no staghorn stones and/or none or mild hydronephrosis such that preventive measures against severe bleeding can be taken to reduce the threat to their lives (Additional file 1).

## Supplementary Information

**Additional file 1: Table S1**. The modified Clavien score.

## Data Availability

The datasets used and/or analyzed during the current study are available from the corresponding author on reasonable request.

## Abbreviations

PCNL: Percutaneous nephrolithotomy
UTIs: Urinary tract infections
EAU: European Association of Urology
CT: Computed tomography
BMI: Body mass index
APD: Anteroposterior diameter
OT: Operative time
ORs: Corresponding odds ratios
CIs: Confidence intervals
ICU: Intensive Care Unit
IQR: Interquartile range
