# Supplementary material for: No staghorn calculi and none/mild hydronephrosis may be risk factors for severe bleeding complications after percutaneous nephrolithotomy
Source: BMC Urol. 2021 Aug 13;21:107. doi: 10.1186/s12894-021-00866-9 (PMC8361647; doi:10.1186/s12894-021-00866-9)
Supplement: Supplementary file 1 — Additional file 1: Table S1. The modified Clavien score. [file 12894_2021_866_MOESM1_ESM.docx]

**Additional file 1**

The modified Clavien score

| Clavien score | - Complication-management definitions |
| --- | --- |
| None | - Normal postoperative trajectory without any unexpected deviation |
|  | - Blocked nephrostomy managed by removal of nephrostomy (without consequences) |
|  | - Nephrostomy tube discomfort that requires removal of nephrostomy |
|  | - Postoperative pain managed by nonopioid analgesics |
| 1  2  3A  3B  4A  4B  5 | - Postoperative pain managed by opioid with or without adjunct analgesic regimen - Postoperative fever (>38.0 C) managed by observation without antibiotics - Deranged renal function that requires IV fluid management only - Bleeding managed using IV fluid without need for blood transfusion - Bleeding that requires a single episode of nephrostomy clamping - Bleeding that requires skin compression/pressure dressing - Renal pelvic perforation managed by watchful waiting - Urine leakage managed by watchful waiting - Ureteric clot managed by watchful waiting - Bladder retention without blood clot that requires bladder catheterization - Pneumothorax managed by watchful waiting - Hydrothorax managed by watchful waiting - Displaced nephrostomy managed by watchful waiting - Intestinal obstruction managed without nasogastric decompression - Bleeding requiring blood transfusion - Nephrostomy site cellulitis managed by antibiotics - Symptomatic UTI managed using antibiotics - Postoperative fever (>38.0 8C) managed with antibiotics in the ward - Colon perforation managed conservatively using IV fluid and antibiotics without controlled colocutaneous fistula - Postoperative ileus managed by nasogastric decompression - Postoperative pneumonia managed by antibiotics - Heart failure (NYHA I and II) requiring management by medications in the ward - Hyposaturation managed by oxygen in the ward - Pulmonary oedema managed by diuretics - Supraventricular arrhythmias requiring antiarrhythmic medications - Minor atelectasis requiring medical management - Febrile UTI or suspected sepsis without organ failure requiring supportive therapy and enhanced monitoring - Bleeding requiring multiple bladder washouts/irrigations - Bleeding managed with haemostatic agents placed endoscopically - Bleeding that requires multiple episodes of nephrostomy clamping (>4 h apart) - Bleeding managed by postoperative ureteric stenting without general anaesthesia - Bleeding managed by postoperative placement of new larger-bore nephrostomy tamponade - Colon perforation managed conservatively using controlled colocutaneous fistula - Hemothorax managed by intercostal draining under local anaesthesia - Hydrothorax managed by intercostal draining under local anaesthesia - Pneumothorax managed by intercostal draining under local anaesthesia - Renal pelvic perforation managed by prolonged nephrostomy tube or postoperative placement of nephrostomy - Renal pelvic perforation managed by ureteric stenting without general anaesthesia - Ureteric clot obstruction managed by ureteric stenting without general anaesthesia - Urine leakage managed by postoperative placement of a new nephrostomy tube - Urine leakage managed by ureteric stenting without general anaesthesia - Blocked nephrostomy managed by ureteric stenting without general anaesthesia - Misplaced double-J stent managed by repositioning - Displaced nephrostomy requiring ureteric stenting without general anaesthesia - Perirenal abscess managed by percutaneous drainage - Bleeding managed by angioembolisation - Bleeding managed by nephrectomy - Colon perforation managed by colostomy - Ureteric stricture managed by balloon dilation - Avulsion of the ureteropelvic junction managed by surgical repair - Retained nephrostomy requiring removal under anaesthesia - Intestinal obstruction managed by gastrostomy - Perirenal abscess managed by open drainage - Bleeding (hypovolaemic shock) requiring ICU management - Adult respiratory distress syndrome requiring ICU management - Hyposaturation requiring ICU management - Pulmonary oedema requiring ICU management - Heart failure requiring ICU management - Hypothermia requiring ICU management - Acute renal failure requiring ICU management - Arrhythmias with haemodynamic instability requiring ICU management - Severe atelectasis requiring intubation and requiring ICU managemen - Urosepsis with multiple organ failure requiring ICU management - Any complication leading to death |

*IV* intravenous, *UTI* urinary tract infection, *NYHA* New York Heart Association, *ICU* intensive care unit.

de la Rosette JJMCH, Opondo D, Daels FPJ, et al. Categorisation of complications and validation of the Clavien score for percutaneous nephrolithotomy. European urology 2012;62(2): 246–255.
